# Clarifying species identity in *Aphanopus* using wavelet-based otolith shape analysis

**DOI:** 10.1371/journal.pone.0326199

**Published:** 2025-06-18

**Authors:** Joana Vasconcelos, Javier Martinez, Airam Guerra-Marrero, José Otero-Ferrer, Alba Jurado-Ruzafa, Ricardo Sousa, Carlos Hernández-González, Mafalda Freitas, Víctor M. Tuset

**Affiliations:** 1 Grupo en Biodiversidad y Conservación (BIOCON), ECOAQUA, Universidad de Las Palmas de Gran Canaria, Canary Islands, Spain; 2 MARE - Marine and Environmental Sciences Centre/ARNET - Aquatic Research Network, Agência Regional para o Desenvolvimento da Investigaç ao Tecnologia e Inovaç ao (ARDITI), Funchal, Portugal; 3 Biostatech, Advice, Training and Innovation in Biostatistics (Ltd.), Santiago de Compostela, Spain; 4 Centro Oceanográfico de Canarias (IEO-CSIC), Santa Cruz de Tenerife, Spain; 5 Direção de Serviços de Investigação, Direção Regional de Pescas, Funchal, Portugal; 6 Instituto de Oceanografía y Cambio Global, IOCAG, Universidad de Las Palmas de Gran Canaria, Unidad Asociada ULPGC-CSIC, Canary Islands, Spain; University of Messina, ITALY

## Abstract

Accurate species identification is crucial for effective fisheries management, particularly for cryptic species with overlapping ranges and similar morphologies. This study explores the coexistence and distribution of *Aphanopus carbo* and *Aphanopus intermedius* in the northeastern Atlantic over four decades using otolith contour analysis. Otolith samples were collected from Madeira and the African coast between 1990 and 2021 and analyzed using a wavelet-based method, which improves species discrimination by capturing finer morphological details. The analysis revealed stable species proportions over time, with *A. carbo* generally dominating the catches (~55–60%), except in 2010 when a decline was observed. A higher presence of *A. intermedius* in offshore areas may be associated with increased salinity near Madeira Island during the spawning season (October–December). Environmental changes, including variations in temperature and salinity at depths greater than 800 m, and the progressive expansion of the drifting longline fishery to new grounds, likely influenced these patterns. This study demonstrates that combining otolith contour analysis with genetically identified reference specimens enhances species discrimination and provides valuable insights into population dynamics and habitat use. These findings contribute to more effective fisheries management and stock assessments for these economically important scabbardfish in the northeastern Atlantic.

## Introduction

Sustainable fishery management relies on accurate assessments that integrate species life cycles, continuous monitoring of fishing activities, and comprehensive fisheries studies [1]. Among the main challenges in such assessments is the presence of cryptic species - groups of genetically distinct organisms that are morphologically similar and have historically been classified under a single species name. These overlooked taxa can mask true biodiversity and lead to inaccurate stock assessments, potentially undermining species-specific conservation and management measures [2]. Misidentification can lead to the overexploitation of sympatric populations due to inaccurate estimates of exploitation rates derived from catch data [3,4]. Genetic approaches, including interspecies distances, molecular markers, and genetic models, have proven valuable for species identification [5], but they can be time-consuming, unlike the proposed methodology. While classical taxonomy remains useful, its labor-intensive nature limits its applicability in fisheries science. An alternative is the integration of genetic and morphological approaches, which improves identification accuracy [6] but still faces challenges in large-scale applications. Otolith contour analysis has been widely used over the past decades as a reliable and cost-effective method for species discrimination, particularly among cryptic and sympatric species. It has successfully distinguished closely related taxa such as *Stellifer* spp. [7] and *Macrourus* spp. [8], among others. In addition to species identification, this method has also been applied to detect hybrids and study convergence patterns associated with environmental changes, including those linked to climate change [9].

A notable example of such a challenge is represented by the commercially important black (*Aphanopus carbo* Lowe, 1839) and intermediate (*A. intermedius* Parin, 1983) scabbardfish — two sympatric species distributed throughout the Atlantic Ocean. Studies confirm their coexistence in the Macaronesian archipelagos and along the coasts of Morocco and Western Sahara, which represent the northernmost range of *A. intermedius*, a species primarily found in tropical and subtropical Atlantic waters [10–12]. These species do not complete their life cycle in a single area. Spawning grounds are located in waters under the jurisdiction of the Fishery Committee for the Eastern Central Atlantic (CECAF), such as those around Madeira and the Canary Islands, whereas juveniles are recruited in northern regions [13–16]. This migratory behaviour, involving both small- and large-scale movements, underscores their vulnerability to fishing pressures. Mature adults migrate to the coastal waters of Madeira (<12 nautical miles, n.m.; 1 n.m. = 1.852 km) during the spawning season [17], making them particularly susceptible to the drifting longline fishery. This practice, which dates back to the $17^{\text{th}}$ century [18], targets their daily feeding movements at depths of 800 to 1,300 m [12,19]. The Madeira fishery accounts for a significant proportion of global landings, with *A. carbo* also being economically valuable in ICES subareas V, VI, VII, VIII, and IX, highlighting the need for effective species identification to support sustainable management [12,16,17]. However, *A. carbo* and *A. intermedius* were historically misclassified as a single species due to the absence of distinct external morphological features [10]. This misclassification persisted until 2010, when Stefanni *et al*. [20] developed a genetic method to distinguish these two cryptic deep-sea scabbardfish species. Subsequently, Biscoito *et al*. [11] integrated external (fin formulae) and internal (vertebral formulae) morphological characteristics to describe *A. intermedius* and differentiate it from *A. carbo* based on prior genetic evidence. In parallel, otolith contour analysis using elliptic Fourier descriptors (EFD) achieved classification rates exceeding 85%, demonstrating its utility for species identification [21]. These studies revealed, for the first time, the broader distribution of both species across the North and Central Atlantic. Despite these advancements, biological studies on *Aphanopus* in the NE Atlantic are often based on mixed-species samples, complicating species-specific assessments. Exceptions include recent analyses of Madeira drifting longline catches [17,22]. Additionally, evidence suggests that *A. carbo* and *A. intermedius* not only exhibit differences in growth traits [23] but also occupy distinct spatial niches, with *A. carbo* inhabiting deeper waters [22,24].

This study aims to access the presence of *Aphanopus carbo* and *A. intermedius* in otolith samples collected over a 40-year period from Madeira landings, as well as in selected samples from Mauritania and Morocco. To achieve this objective, otolith contour shape was analysed using wavelet-based methods [25], which outperform elliptic Fourier descriptors (EFD) by enhancing both biological and mathematical interpretation [26], thereby providing excellent species discrimination [27,28]. Based on previous studies, we hypothesise that *Aphanopus carbo* and *A. intermedius* have co-occurred historically in the northeastern Atlantic and that wavelet-based otolith contour analysis, trained on genetically identified specimens, provides a reliable tool for reconstructing species composition from historical collections.

## Materials and methods

### Ethics statement

No permits were required for the described study, as otoliths were obtained from samples captured by commercial fishery operations.

### Data collection

Otolith contours from 136 individuals of *Aphanopus carbo* (N = 70) and *Aphanopus intermedius* (N = 66), previously genetically identified using mitochondrial COI and 16S rRNA sequences by Biscoito *et al*. [11], were analysed for shape variation. These specimens originated from commercial and experimental fishing in mainland Portugal, the Azores, Madeira, the Canary Islands, Morocco, and Western Sahara (S1 Table; Fig 2). This reference dataset was used to train the wavelet-based classification model.

**Fig 1:**
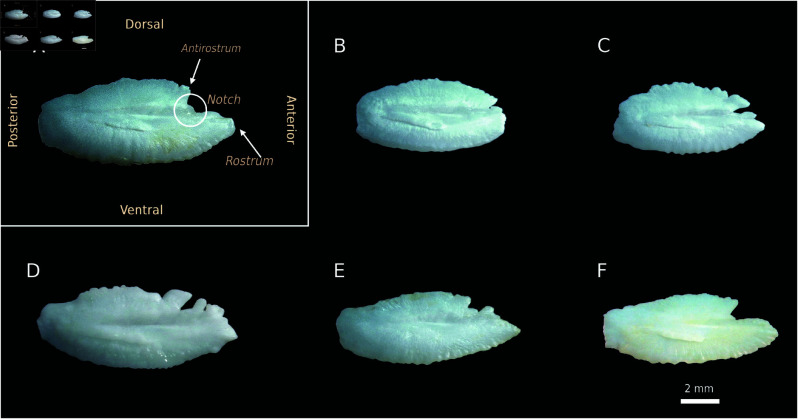
Otoliths from specimens of Aphanopus carbo (A–C) and A. intermedius (D–F), identified based on meristic characters. Species identification was supported by vertebral and dorsal spine counts following Biscoito *et al*. [11]. All specimens were collected from the Madeira Exclusive Economic Zone. Capture years: A–C and E–F (2021); D (2010). Otolith lengths (OL): A, 10.0 mm; B, 7.6 mm; C, 7.7 mm; D, 10.0 mm; E, 9.4 mm; F, 8.9 mm. Images show the medial (inner) side of each otolith.

**Fig 2:**
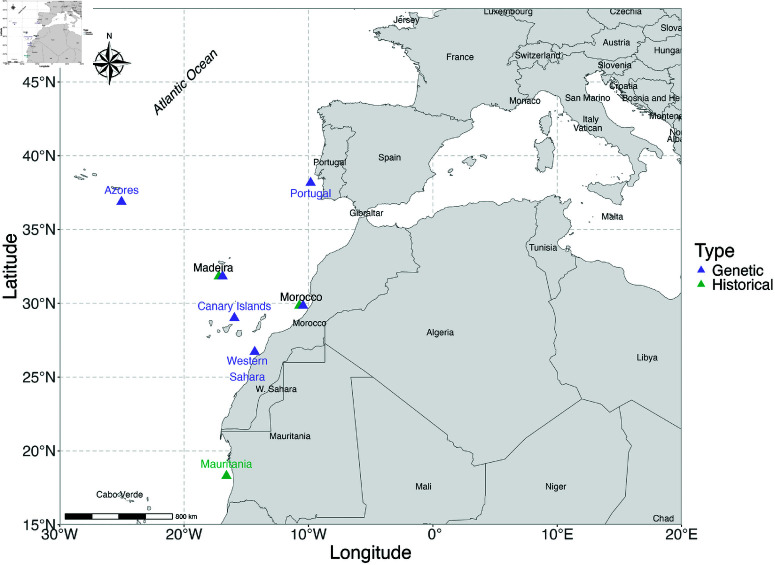
Locations of historical (green triangles) and genetic (purple triangles) sampling of Aphanopus carbo and Aphanopus intermedius across the northeastern Atlantic. Shared sampling sites (Madeira and Morocco) are shown as offset triangles and labelled in black. Genetic samples were obtained from published, genetically identified specimens [11], while historical samples correspond to otoliths collected from Madeiran landings between 1990 and 2021, and from research surveys off Morocco and Mauritania.

This model was then applied to a set of 1,975 otoliths collected from Madeiran commercial longliners in 1990, 2003, 2010, and 2021 to reconstruct the historical species composition of the landings. From 2005 onward, the fishery expanded in search of new fishing grounds beyond the traditional areas, reflecting a progressive geographical expansion of the deep-sea longline fleet [12,22]. These specimens were stored in the Fisheries Regional Directorate in Madeira. Before 2010, all specimens were classified as *A. carbo*, whereas post-2010 identifications followed the morphological and meristic criteria of Biscoito *et al*. [11]. Additionally, we included 547 specimens from Mauritania and Morocco, obtained during IEO-CSIC trawling surveys (2005-2007) at depths greater than 500 meters, which, like the early Madeira samples, were classified as *A. carbo*.

Temperature and salinity data at 800 m depth were extracted from the Copernicus GLORYS12V1 dataset (https://doi.org/10.48670/moi-00021), which provides sea water potential temperature (thetao) and practical salinity for the period 1993–2021. Potential temperature represents the temperature seawater would have if brought adiabatically to the surface, allowing consistent comparisons across depths and time. However, to illustrate conditions in 1990—prior to the GLORYS12V1 time range—in-situ temperature at 800 m was obtained from the CORA v5.2 dataset (https://doi.org/10.17882/46219).

### Contour otolith analysis

Left otoliths were positioned with the inner side (*sulcus acusticus*) facing upward and the *rostrum* oriented to the right, against a black background, for digital imaging. Photographs were captured using digital cameras attached to a stereomicroscope, with a fixed magnification of 6.7×. Otolith length (OL, measured to 0.01 mm) was determined using ImageJ software [29].

The shape contour analysis employed a wavelet function developed by the AFORO team to identify local morphological variation along the x-axis of the otolith contour [26,28]. A total of 512 equidistant Cartesian coordinates were extracted from each otolith projection, with the anterior tip of the *rostrum* used as the starting point. These coordinates were then analysed using wavelet transformed (WLT; see Parisi-Baradad *et al*. [25]). Of the nine detail levels produced by the wavelet function, the 5th level was selected as optimal for distinguishing interspecific variability [30].

### Statistical analysis

To evaluate differences in mean otolith length (OL, mm) among samples (species or regions), a Kruskal-Wallis (KW) test was employed since the data violated normality (Shapiro–Wilk test, P < 0.05) and homogeneity of variance (Levene test, P < 0.05).

Wavelet transform coefficients were subjected to a Principal Component Analysis (PCA) to reduce dimensionality and extract the major axes of shape variation. The number of informative components retained for further analysis was determined using the ‘broken stick model’ [31], which compares observed eigenvalues to those expected under a null distribution. To account for the potential influence of otolith size, we performed Pearson’s correlations between each retained PC and otolith length (OL). The residuals from these correlations—representing shape variation independent of size—were used in a second PCA, which served as the input for classification analyses [32].

Significant total variation was tested using permutational multivariate analysis of variance (PERMANOVA; [33]), employing 9.999 permutations with Manhattan distance. Post-hoc pairwise comparisons were adjusted using Bonferroni correction. To identify patterns and predict species assignment, artificial neural networks (ANNs) were applied using a multi-layer perceptron (MLP) model, calibrated through a back-propagation gradient algorithm [34–36]. The classifications were performed using the R packages *caret* v.6.0.94 [37] and *RSNNS* v.0.4.16 [38]. For the limited genetic sample size (<150 individuals per species), a Leave-One-Out Cross-Validation (LOOCV) approach was adopted [39], iterating each observation as the validation set while training on the remaining data. This process, repeated 1,000 times per analysis, ensured robust classification accuracy and model evaluation. Optimal hyperparameters were fine-tuned during preliminary analyses, with scaled and centred predictor variables. For species assignment of ‘unknown’ samples from Madeira and Africa, the model was trained on the genetic dataset and then applied to the historical samples using the same number of principal components to ensure input compatibility with the MLP structure. All statistical analyses and data processing were carried out using R Statistical Software (v4.4.1; [40]).

## Results

### Otolith-based discrimination of genetic samples

**Fig 3:**
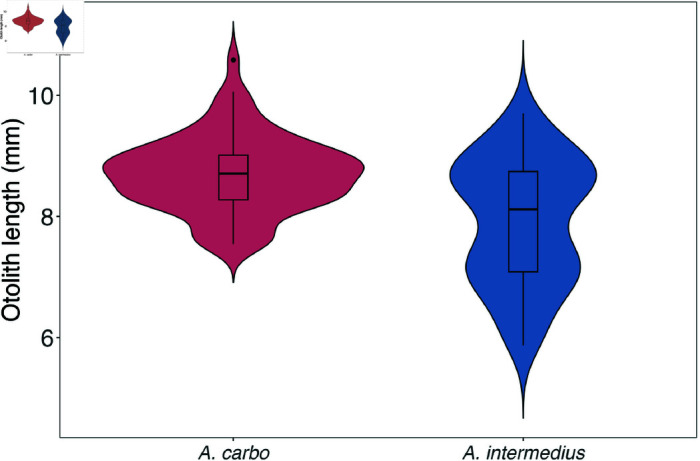
Otolith length variation in genetically identified Aphanopus carbo and A. intermedius from the northeastern Atlantic. Violin plots show the distribution, median, and interquartile range of otolith lengths (in mm) for each species. A total of 136 genetically identified individuals were analysed (70 *A. carbo*, 66 *A. intermedius*), sampled from Madeira, Canary Islands, and Western Sahara.

**Fig 4:**
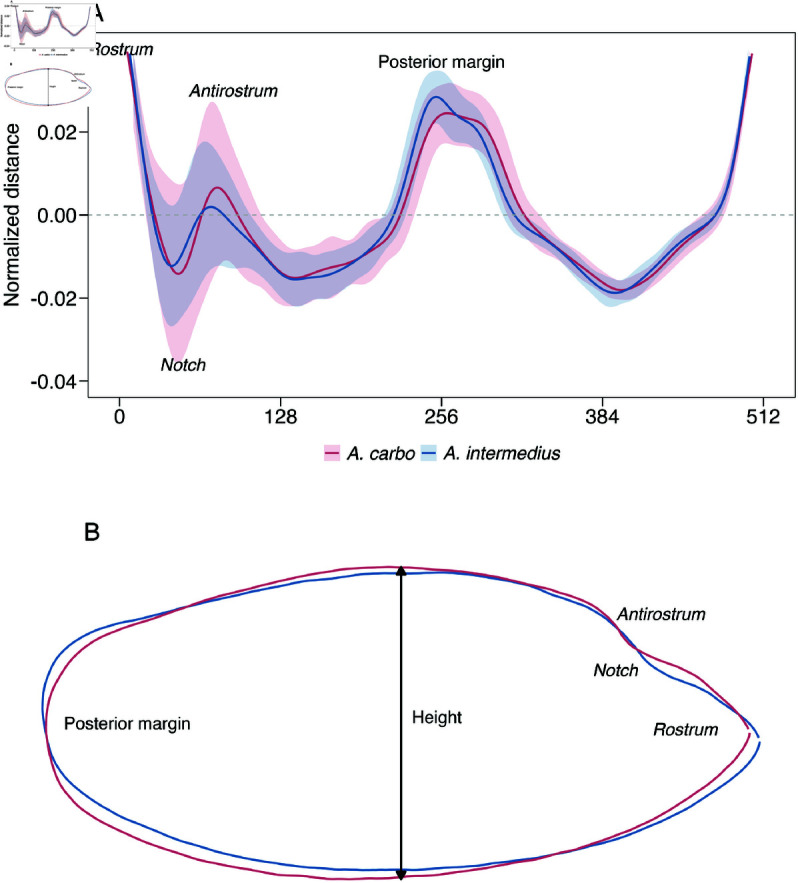
Mean otolith shape of genetically identified Aphanopus carbo (red) and A. intermedius (blue). Reconstructed using wavelet analysis to represent the average phenotype from the northeastern Atlantic. (A) Mean otolith contour (solid line) with standard deviation (shaded area), highlighting regions with higher intraspecific variability. The contour is based on the $5^{\text{th}}$ wavelet decomposition. The X-axis represents 512 equidistant points along the otolith perimeter, while the Y-axis represents the mean normalized distance. (B) Reconstructed mean otolith shape based on coordinate data.

**Fig 5:**
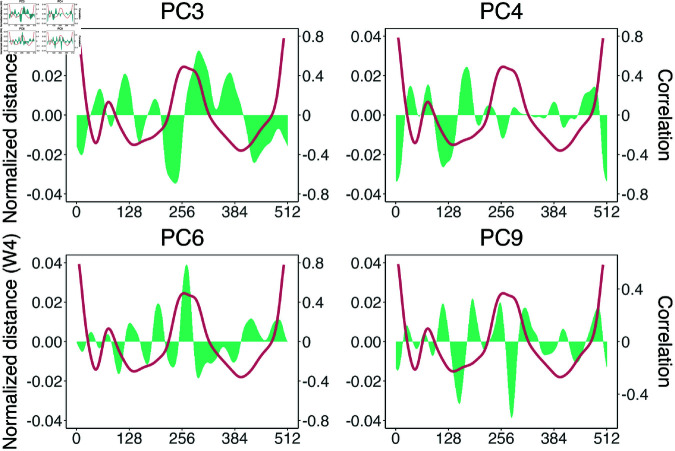
Correlation between the most relevant principal components (PCs) for classification and the normalized distance of the $5^{\text{th}}$-level wavelet decomposition. Shown are PCs 3, 4, 6, and 9, identified as the most informative for species classification based on variable importance scores from a multilayer perceptron model. Green areas represent PC loading patterns, and the red line shows the normalized $5^{\text{th}}$-level wavelet of a representative *Aphanopus carbo* specimen. These components highlight key shape features that distinguish between *A. carbo* and *A. intermedius*, sampled from the northeastern Atlantic.

Significant interspecific differences in the OL variable were detected (Wilcox test, W= 3288, p < 0.001) on the genetic sample, with *A. carbo* exhibiting higher values (Fig 3; S1 Table). The first nine principal components (PCs), selected using the broken stick model, explained 88.8% of the total variance (S2 Table).The PERMANOVA analysis revealed significant differences in otolith shape between species (F1,135 = 2.890, p = 0.006). Wavelet functions (Fig 4A) and otolith contours (Fig 4B) showed that, while both species shared a broadly similar average otolith pattern, notable differences were observed in three key regions: (i) anterior margin, where *A. carbo* displayed a more pronounced *antirostrum* and a deeper *notch* compared to *A. intermedius* which exhibited an almost absent *antirostrum* and a shallower *notch*; (ii) height, with *A. intermedius* having thinner and more elongated otoliths, whereas *A. carbo* showed wider and more elliptic shapes; and (iii) posterior margin, where *A. carbo* otoliths were more rounded, contrasting with the oblique posterior margin characteristic of *A. intermedius*. When considering the average phenotype per species, the classification accuracy was 94.1%, with a Cohen’s kappa of 0.882 (Table 1). The high classification accuracy was primarily driven by shape variation described by PC3, PC4, PC6, and PC9 (Fig 5). PC3 was associated with curvature in the postero-dorsal and postero-ventral regions, PC4 reflected variation in *rostrum* length and overall otolith height, PC6 captured signal from the posterior margin, and PC9 associated with changes concentrated in the dorsal-posterior contour.

**Table 1:** Confusion/error matrix (LOOCV) showing phenotype abundances, classification accuracy, and the kappa index obtained using a multi-layer perceptron (MLP) classifier for *Aphanopus carbo* and *A. intermedius*. Individuals were genetically identified and sampled from the northeastern Atlantic, including mainland Portugal, the Azores, the Madeira Archipelago, Morocco, and Mauritania. Correctly predicted group memberships are highlighted in bold.

	References		Performance measures		
Prediction	*A. carbo*	*A. intermedius*	Accuracy	Kappa	% Accuracy
A. carbo	**68**	6			97.14
A. intermedius	2	**60**			90.90
*Total*	70	66	0.9412	0.882	

#### Reconstructing historical species assignments

In the Madeira dataset, the first 15 principal components exceeded the broken-stick threshold, explaining 96.0% of the total shape variance (S3 Table). However, since the MLP classification model was trained using only the first 9 PCs from the genetic dataset, species assignment in historical samples was likewise based on the first 9 components, which accounted for 88.7% of the variance. For the African dataset, the first 15 PCs explained 95.5% of the variance, with the first 9 covering 87.0% (S4 Table).

**Fig 6:**
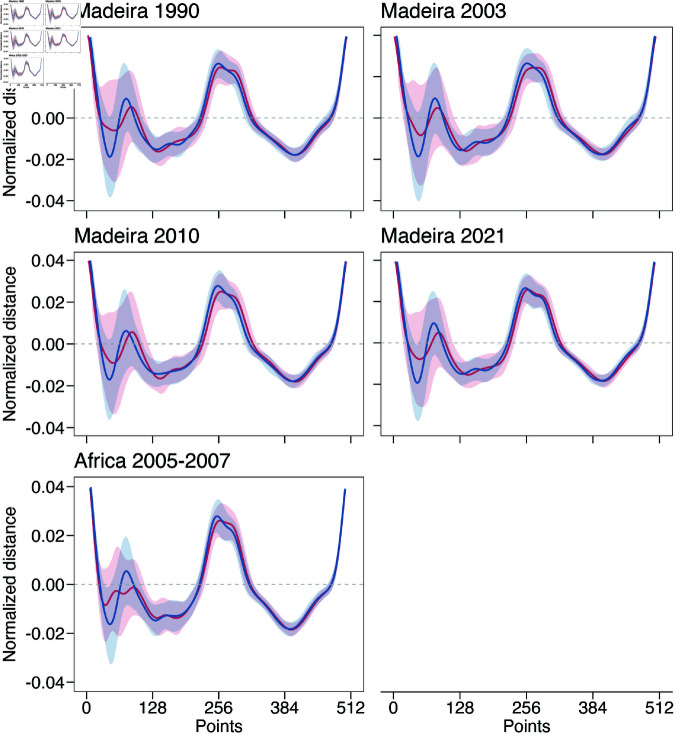
Mean otolith contour (solid line) with standard deviation (shaded area) of the wavelet decomposition for samples collected from Madeira across four decades (1990–2021) and the African coast (Morocco and Mauritania, 2005–2007). The contour is based on the 5th wavelet decomposition. The X-axis represents 512 equidistant points along the otolith perimeter, while the Y-axis represents the mean normalized distance.

The predictive model estimated a mean ratio of 1.18:1 for *A. carbo* to *A. intermedius* in Madeira samples, with the highest presence of *A. carbo* in 2003 (1.50:1) and the lowest in 2010 (0.89:1) (Table 2). Model validity was reinforced by the alignment between average otolith patterns (wavelet function) of the genetic sample and the predicted data (Figs 4 and 6). Exceptions included *A. intermedius* otoliths from Madeira and the African coast, which displayed a more elongated *rostrum* and a more pronounced *antirostrum* compared to *A. carbo* (Fig 6).

**Table 2:** Number (N) of otoliths, and their corresponding mean and standard deviation (SD) of otolith length (in mm), classified as *Aphanopus carbo* and *A. intermedius*. Classification results obtained using the MLP model applied to historical samples from Madeira, Morocco, and Mauritania.

Location	Year	*A. carbo*		*A. intermedius*	
		N (%)	Mean ± SD (range)	N (%)	Mean ± SD (range)
Madeira	1990	282 (54)	8.81 ± 0.63 (6.7 – 10.5)	242 (46)	8.85 ± 0.68 (5.3 – 11.1)
	2003	293 (60)	8.98 ± 0.58 (7.5 – 10.8)	201 (40)	9.00 ± 0.63 (7.7 – 10.9)
	2010	227 (47)	8.71 ± 0.65 (7.3 – 10.8)	251 (53)	8.76 ± 0.64 (7.2 – 10.6)
	2021	266 (56)	8.66 ± 0.60 (6.7– 10.3)	213 (44)	8.73 ± 0.58 (7.3 – 10.3)
Morocco	2005	216 (52)	7.89 ± 1.06 (6.0 – 10.3)	202 (48)	7.87 ± 1.03 (6.0 – 10.2)
Mauritania	2007	67 (52)	6.72 ± 0.48 (6.0 – 8.5)	62 (48)	6.90 ± 0.59 (6.0 – 9.7)

## Discussion

This study establishes wavelet transform analysis as a powerful tool for differentiating cryptic species, achieving high accuracy (94.1%) in classifying *Aphanopus carbo* and *A. intermedius*. By identifying species-specific morphological traits and reconstructing their distribution over time, our findings address key knowledge gaps in the local management of these economically important scabbardfish, particularly in the Madeira region within the CECAF area. Validation of wavelet-based contour shape analysis against genetically identified specimens enhances classification precision and provides a scalable approach for analysing extensive historical datasets, offering insights into population dynamics over four decades. The wavelet transform method effectively captured subtle morphological differences, particularly in localized otolith regions such as the anterior margin, *antirostrum*, posterior margin, and general height, which contributed to the separation of *A. carbo* and *A. intermedius*. The high classification accuracy (94.1%) and strong Cohen’s kappa (0.882) underscore the reliability of this approach. While methodological differences limit direct comparisons, these results suggest that wavelet-based analysis can achieve higher discriminatory performance than previous applications of Fourier descriptors in similar contexts [21].

Applying this method to historical datasets from Madeira revealed consistent species proportions over four decades (1990, 2003, 2010, and 2021), with both species being regularly captured. This contradicts previous suggestions that *A. intermedius* is restricted to the South Atlantic. *A. carbo* generally dominated the catches (*ca.* 55—60%), except in 2010, when a decline was observed. However, these temporal patterns are not conclusive regarding the abundance and distribution of both species, as they may reflect a combination of environmental changes, fishing pressure, species-specific life history traits, and the progressive geographical expansion of the drifting longline fishery to new areas—including the Southern Azores Seamount Chain and the Canary Islands’ Exclusive Economic Zone—as reported in previous studies driven by declining catch weights around Madeira [12,17]. The increased proportion of *A. intermedius* in offshore catches (>12 nm) in 2010 aligns with the shift of the Madeiran fleet toward more distant fishing grounds, such as the Canary Islands’ ZEE, where *A. intermedius* tends to dominate [22]. Moreover, species distribution also varies with seafloor depth, with *A. carbo* more frequently caught in shallower fishing grounds, while *A. intermedius* is more dominant in deeper fishing areas—a pattern consistent under niche-based hypothesis to reduce interspecific competition [22]. The limited knowledge of their ecology and biology makes it difficult to understand why their coexistence has remained stable over time.

**Fig 7:**
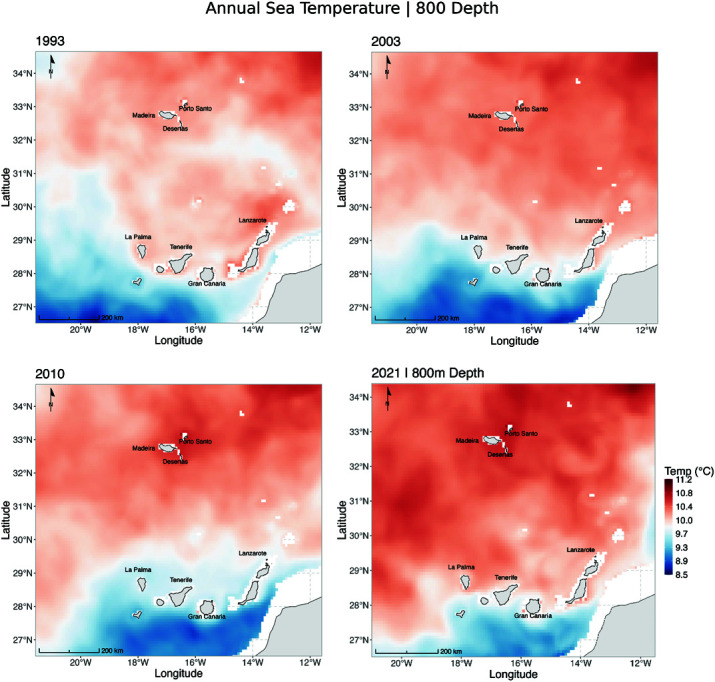
Annual sea temperature (∘C) at 800 m depth in the Madeira and Canary Islands region for the years 1993, 2003, 2010, and 2021. Warmer temperatures are represented in shades of red, while cooler temperatures are shown in shades of blue. The maps highlight an overall warming trend over the decades, particularly near Madeira Island and the northernmost areas.

**Fig 8:**
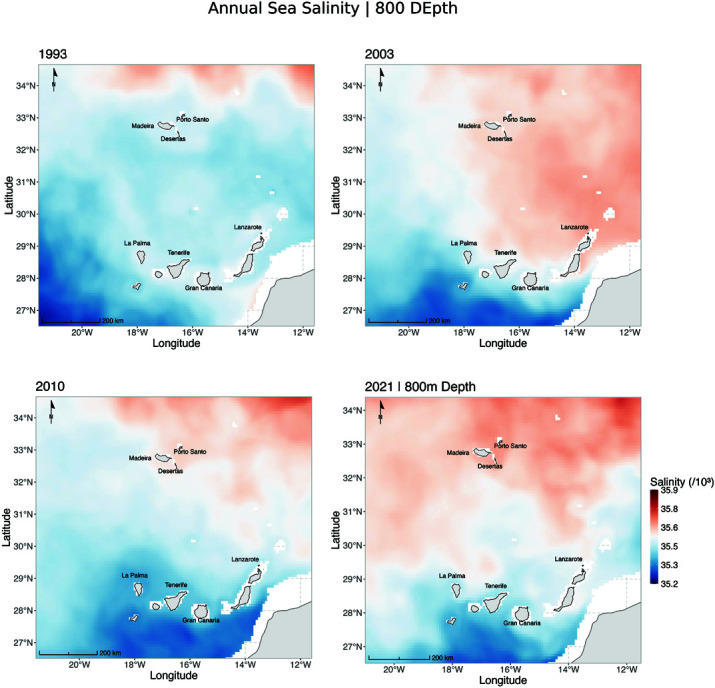
Annual sea salinity ($\times 10^{3}$) at 800 m depth in the Madeira and Canary Islands region for the years 1993, 2003, 2010, and 2021. Higher salinity values are represented in shades of red, while lower salinity values are shown in shades of blue. The maps highlight an increase in salinity over the decades, particularly in the northern areas near Madeira Island.

**Fig 9:**
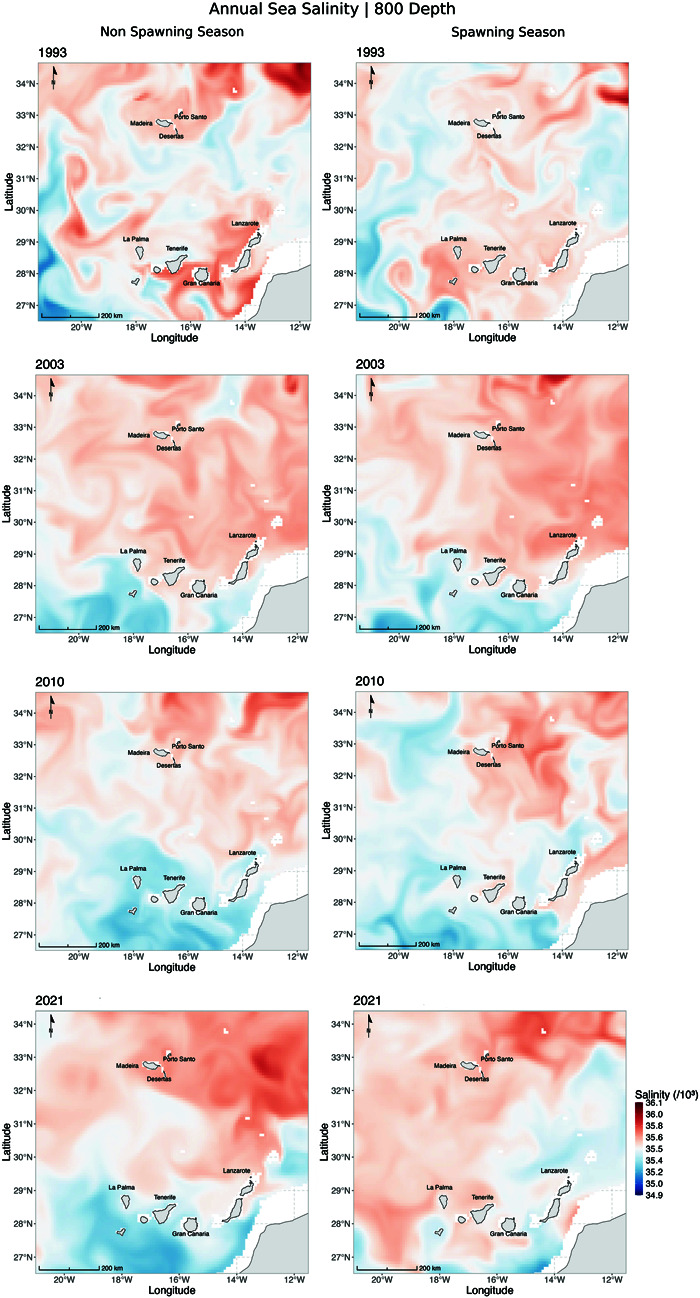
Annual sea salinity ($\times 10^{3}$) at 800 m depth in the Madeira and Canary Islands region for the years 1993, 2003, 2010, and 2021. Comparing the non-spawning season (left) and spawning season (right). Higher salinity values are represented in shades of red, while lower salinity values are shown in shades of blue. The maps highlight consistently higher salinity near Madeira Island during the spawning season (October–December).

Environmental changes, particularly variations in temperature and salinity at depths greater than 800 m, may have influenced species distribution patterns over time (Figs 7–9, S1 and S2 Figs). Salinity, in particular, affects the buoyancy of marine fish eggs, influencing their vertical positioning in the water column—a critical factor for larval survival and development [44]. These effects are especially relevant during the spawning season (October–December, Fig 9), when most fishing occurs within territorial waters, particularly in submarine canyons and along adjacent slopes near Madeira Island [17]. The Mediterranean Outflow Water (MOW), a dense and saline water mass originating from the Strait of Gibraltar, flows into the North Atlantic through three main branches: a westward branch toward the central Atlantic, a poleward branch along the western European margin, and a southwestward branch along the African continental slope, potentially reaching Madeira and the Canary Islands [41–43]. This water mass may influence deep-sea hydrographic conditions relevant to spawning. Interestingly, a higher proportion of *A. carbo* (60%) was observed in 2003, coinciding with an anomaly of low salinity in the western MOW branch [43]. In contrast, 2010 showed particularly low salinity values between Madeira and the Canary Islands—the same year when *A. intermedius* was most abundant in the landings. While direct causality cannot be inferred, these patterns suggest that temporal variability in salinity may interact with life history traits to affect reproductive success and, ultimately, species composition in fisheries catches.

## Conclusion

While this study highlights the effectiveness of wavelet-based otolith contour analysis for species classification, certain limitations should be considered. The relatively small number of genetically identified specimens per region may have influenced the predicted proportions of *A. carbo* and *A. intermedius* over time. Expanding the genetic dataset would improve model training and enhance the accuracy of species identification across both temporal and spatial scales.

Although the model achieved high classification accuracy on genetically identified specimens collected in the mid 2000s, applying it to historical otoliths from other decades may introduce uncertainty. Environmental changes over time may influence otolith morphology, and further validation using temporally stratified genetic data would help assess the robustness of shape-based classification across time. In addition, because our analysis is based on four non-consecutive sampling years (1990, 2003, 2010, and 2021), it does not capture potential interannual cycles or trends in species proportions. The results should therefore be interpreted as snapshots rather than continuous temporal patterns.

A holistic approach that combines genetic data, morphometric analyses, and environmental factors would enhance our understanding of population structure and habitat connectivity, paving the way for more effective and sustainable management strategies.

## Supporting information

S1 Fig In-situ sea temperature (∘C) and salinity (psu) at 800 m depth in the Madeira and Canary Islands region for the year 1990, based on CORA v5.2. The left panel shows temperature and the right panel shows salinity. Warmer temperatures and higher salinity are represented in red tones, while cooler and fresher values appear in blue. Grid smoothing was applied to reduce artifacts due to sparse data coverage, particularly near islands and continental slopes. These maps provide an indicative overview of oceanographic conditions at the onset of the study period, but are not directly comparable to GLORYS12V1-based maps of potential temperature.
(TIFF)

S2 Fig Annual sea temperature (∘C) at 800 m depth in the Madeira and Canary Islands region for the years 1993, 2003, 2010, and 2021. Comparing the non-spawning season (left) and spawning season (right). Warmer temperatures are represented in shades of red, while cooler temperatures are shown in shades of blue. The maps highlight a general warming trend over the decades, with particularly elevated temperatures near Madeira Island during the spawning season in 2020 compared to the non-spawning season.
(TIFF)

S1 Table Number of individuals, mean otolith length (OL, mm), and standard deviation (SD) for *Aphanopus carbo* and *A. intermedius* samples collected across the northeastern Atlantic. These include mainland Portugal, the Azores, Madeira Archipelago, Morocco, and Western Sahara.
(PDF)

S2 Table Variance explained by principal components derived from otolith shape analysis of genetically identified *Aphanopus carbo* and *A. intermedius* samples. Collected across the northeastern Atlantic, including mainland Portugal, the Azores, the Madeira Archipelago, Morocco, and Western Sahara.
(PDF)

S3 Table Variance explained by principal components derived from otolith shape analysis of Aphanopus carbo and A. intermedius samples. The samples were collected in Madeira.
(PDF)

S4 Table Variance explained by principal components derived from otolith shape analysis of *Aphanopus carbo* and *A. intermedius* samples. The samples were collected from Morocco and Western Sahara (African samples).
(PDF)
