# Supplementary figures and images for: Clarifying species identity in Aphanopus using wavelet-based otolith shape analysis
Source: PLoS One. 2025 Jun 18;20(6):e0326199. doi: 10.1371/journal.pone.0326199 (PMC12176198; doi:10.1371/journal.pone.0326199)

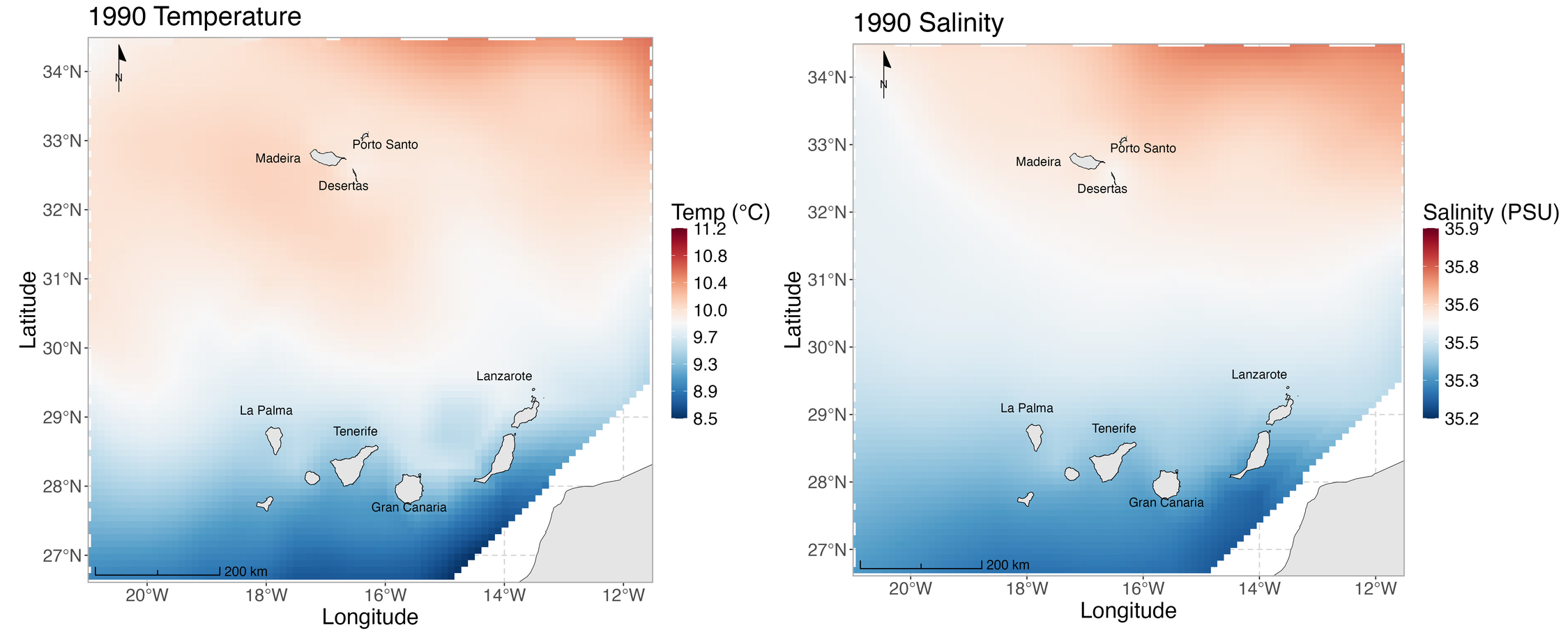

Supplement: S1 Fig — The left panel shows temperature and the right panel shows salinity. Warmer temperatures and higher salinity are represented in red tones, while cooler and fresher values appear in blue. Grid smoothing was applied to reduce artifacts due to sparse data coverage, particularly near islands and continental slopes. These maps provide an indicative overview of oceanographic conditions at the onset of the study period, but are not directly comparable to GLORYS12V1-based maps of potential temperature. (TIFF) [file pone.0326199.s001.tif]

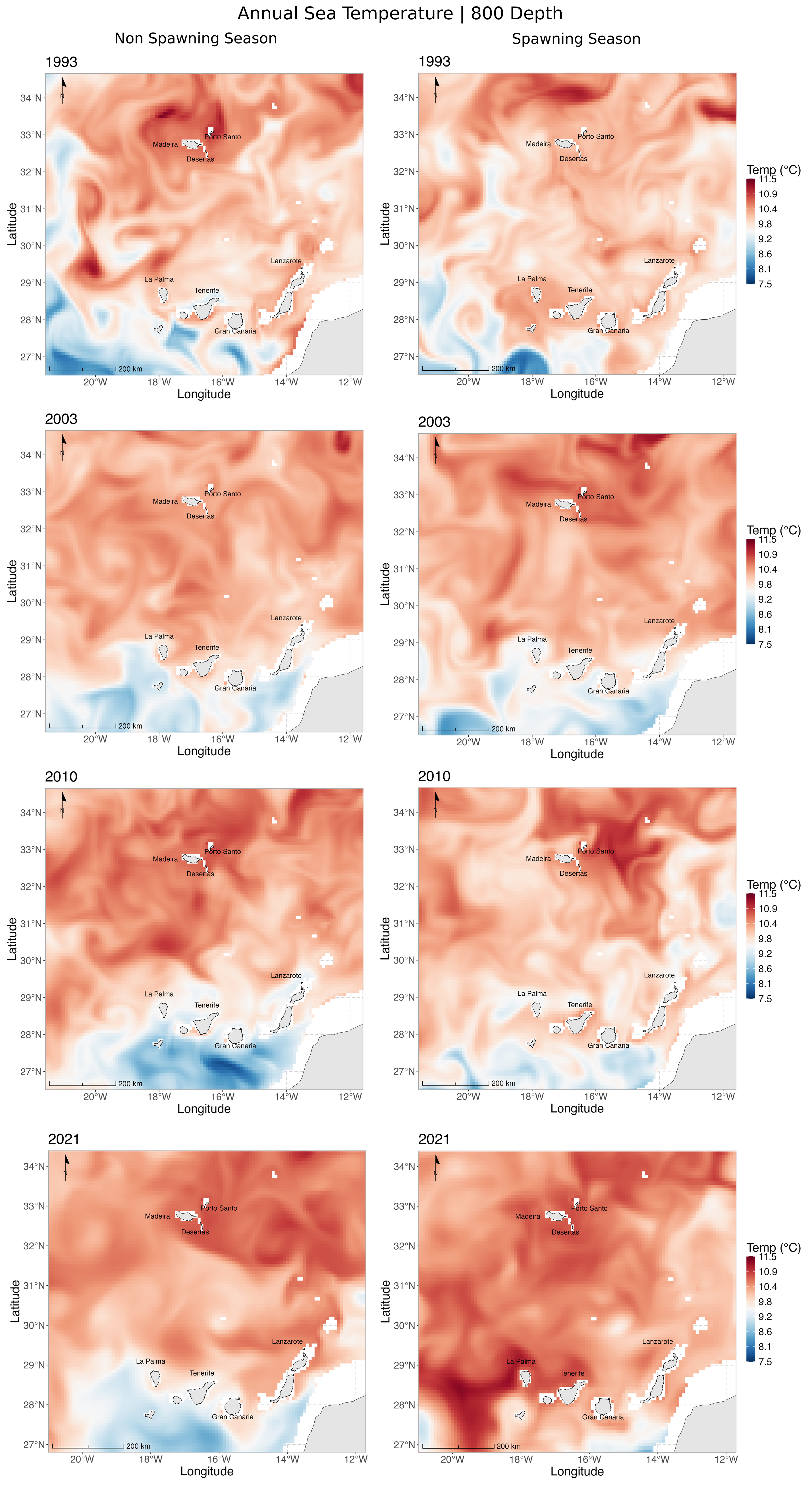

Supplement: S2 Fig — Comparing the non-spawning season (left) and spawning season (right). Warmer temperatures are represented in shades of red, while cooler temperatures are shown in shades of blue. The maps highlight a general warming trend over the decades, with particularly elevated temperatures near Madeira Island during the spawning season in 2020 compared to the non-spawning season. (TIFF) [file pone.0326199.s002.tif]
