# Supplementary material for: Clarifying species identity in Aphanopus using wavelet-based otolith shape analysis
Source: PLoS One. 2025 Jun 18;20(6):e0326199. doi: 10.1371/journal.pone.0326199 (PMC12176198; doi:10.1371/journal.pone.0326199)
Supplement: S1 Table — These include mainland Portugal, the Azores, Madeira Archipelago, Morocco, and Western Sahara. (PDF) [file pone.0326199.s003.pdf]

1.2 Tables

S1 Table.. **Number of individuals, mean otolith length (OL, mm), and standard deviation (SD) for *Aphanopus carbo* and *A. intermedius* samples collected across the northeastern Atlantic.** These include mainland Portugal, the Azores, Madeira Archipelago, Morocco, and Western Sahara.

		A. carbo			*A. intermedius*		
Method	Locality/Year	N	Mean ± SD	Min. - Max.	N	Mean ± SD	Min. - Max.
Genetically identified	Mainland Portugal	25	8.39 ± 0.483	7.54 - 9.25	-	-	-
	Azores	3	8.66 ± 0.362	8.29 - 9.01	26	8.20 ± 0.744	6.82 – 9.62
	Madeira	19	9.10 ± 0.657	7.72 – 10.6	7	8.58 ± 0.944	6.73 – 9.51
	Morocco	4	8.19 ± 0.677	7.58 – 9.11	10	7.34 ± 0.798	6.28 – 8.66
	Canary Islands	17	8.85 ± 0.520	8.16 – 10.1	10	8.91 ± 0.439	7.99 – 9.70
	Western Sahara	2	8.52 ± 0.407	8.23 – 8.81	13	6.67 ± 0.555	5.87 – 7.40
	total	70	8.70 ± 0.619	7.54 – 10.6	66	7.92 ± 1.03	5.87 – 9.70
Species identification	Madeira 1990	524	8.83 ± 0.650	5.32 - 11.1	-	-	-
	Madeira 2003	494	8.99 ± 0.600	7.45 – 10.9	-	-	-
	Madeira 2010	478	8.74 ± 0.644	7.22 – 10.8	-	-	-
	Madeira 2021	479	8.69 ± 0.592	6.72 – 10.3	-	-	-
	Morocco	418	7.88 ± 1.04	6.01 – 10.3	-	-	-
	Mauritania	129	6.81 ± 0.543	6.01 – 9.65	-	-	-
