# Supplementary material for: Clarifying species identity in Aphanopus using wavelet-based otolith shape analysis
Source: PLoS One. 2025 Jun 18;20(6):e0326199. doi: 10.1371/journal.pone.0326199 (PMC12176198; doi:10.1371/journal.pone.0326199)
Supplement: S2 Table — Collected across the northeastern Atlantic, including mainland Portugal, the Azores, the Madeira Archipelago, Morocco, and Western Sahara. (PDF) [file pone.0326199.s004.pdf]

S2 Table.. Variance explained by principal components derived from otolith shape analysis of genetically identified *Aphanopus carbo* and *A. intermedius* samples. Collected across the northeastern Atlantic, including mainland Portugal, the Azores, the Madeira Archipelago, Morocco, and Western Sahara.

	**Eigenvalue**	**Variance (%)**	**Cumulative Variance (%)**
PC1	0.0109191	30.45	30.45
PC2	0.0099907	27.86	58.31
PC3	0.0031973	8.92	67.23
PC4	0.0020618	5.75	72.98
PC5	0.0016414	4.58	77.56
PC6	0.0012343	3.44	81.00
PC7	0.0010529	2.94	83.93
PC8	0.0009837	2.74	86.68
PC9	0.0007499	2.09	88.77
