# Supplementary material for: Clarifying species identity in Aphanopus using wavelet-based otolith shape analysis
Source: PLoS One. 2025 Jun 18;20(6):e0326199. doi: 10.1371/journal.pone.0326199 (PMC12176198; doi:10.1371/journal.pone.0326199)
Supplement: S3 Table — The samples were collected in Madeira. (PDF) [file pone.0326199.s005.pdf]

S3 Table.. Variance explained by principal components derived from otolith shape analysis of *Aphanopus carbo* and *A. intermedius* samples. The samples were collected in Madeira.

	**Eigenvalue**	**Variance (%)**	**Cumulative Variance (%)**
PC1	0.0150184	33.15	33.15
PC2	0.0125845	27.78	60.93
PC3	0.0037700	8.32	69.25
PC4	0.0024131	5.33	74.58
PC5	0.0017596	3.88	78.46
PC6	0.0013673	3.02	81.48
PC7	0.0012012	2.65	84.13
PC8	0.0011349	2.51	86.64
PC9	0.0009418	2.08	88.72
PC10	0.0007573	1.67	90.39
PC11	0.0007302	1.61	92.00
PC12	0.0005545	1.22	93.22
PC13	0.0004680	1.03	94.26
PC14	0.0004593	1.01	95.27
PC15	0.0003387	0.75	96.02
