# Supplementary material for: Clarifying species identity in Aphanopus using wavelet-based otolith shape analysis
Source: PLoS One. 2025 Jun 18;20(6):e0326199. doi: 10.1371/journal.pone.0326199 (PMC12176198; doi:10.1371/journal.pone.0326199)
Supplement: S4 Table — The samples were collected from Morocco and Western Sahara (African samples). (PDF) [file pone.0326199.s006.pdf]

S4 Table.. Variance explained by principal components derived from otolith shape analysis of *Aphanopus carbo* and *A. intermedius* samples. The samples were collected from Morocco and Western Sahara (African samples).

	**Eigenvalue**	**Variance (%)**	**Cumulative Variance (%)**
PC1	0.0076519	27.70	27.70
PC2	0.0066651	24.13	51.82
PC3	0.0023876	8.64	60.46
PC4	0.0020503	7.42	67.89
PC5	0.0013128	4.75	72.64
PC6	0.0012484	4.52	77.16
PC7	0.0011239	4.07	81.22
PC8	0.0008372	3.03	84.26
PC9	0.0007630	2.76	87.02
PC10	0.0005846	2.12	89.13
PC11	0.0005596	2.03	91.16
PC12	0.0003701	1.34	92.50
PC13	0.0003458	1.25	93.75
PC14	0.0002815	1.02	94.77
PC15	0.0002067	0.75	95.52
